# How I set up positive end-expiratory pressure: evidence- and physiology-based!

**DOI:** 10.1186/s13054-019-2695-z

**Published:** 2019-12-16

**Authors:** Emanuele Rezoagli, Giacomo Bellani

**Affiliations:** 1 Department of Medicine and Surgery, University of Milan-Bicocca, Monza, Italy; 2 Department of Emergency and Intensive Care, San Gerardo Hospital, Monza, Italy

Positive end-expiratory pressure (PEEP) is a cornerstone treatment for critically ill patients, with beneficial effects for acute respiratory distress syndrome (ARDS).

In ARDS, PEEP prevents alveolar collapse during expiration and counteracts increased surface tension due to surfactant impairment, alveolar over-deflation, and superimposed pressure. These mechanisms contribute to a reduction in intrapulmonary shunting. Furthermore, alveolar recruitment maintained through PEEP may translate into a higher end-expiratory lung volume (EELV), which often leads to better compliance of the respiratory system (C_RS_) and therefore a reduction in the driving pressure (DP), both of which are associated with higher survival rates [1]. Moreover, alveolar stability protects against intra-tidal recruitment/derecruitment (i.e., atelectrauma) [2] and imposed mechanical stresses and inflammation (i.e., biotrauma) [3], and it reduces ventilation heterogeneity [4].

Advantages of PEEP should be balanced against its potential disadvantages, namely, a reduction in cardiac output, an increase in pulmonary vascular resistance and alveolar dead space, and the risk of regional over-inflation [5].

## Recommended PEEP titration

Current guidelines concerning moderate or severe ARDS recommend using higher rather than lower PEEP levels [6]. This recommendation is based on meta-analysis of individual patient data [7]. Furthermore, a subsequent ancillary analysis of LUNG SAFE reported that higher PEEP levels are associated with improved survival [8].

## How do we set up PEEP

We present a PEEP titration strategy that relies heavily on physiological considerations, which is applied at our center. This opinion-based editorial is based on our interpretation of the evidence-based medical literature and on clinical experience, without presumptions of exhaustiveness or superiority to other strategies.

**Fig. 1:**
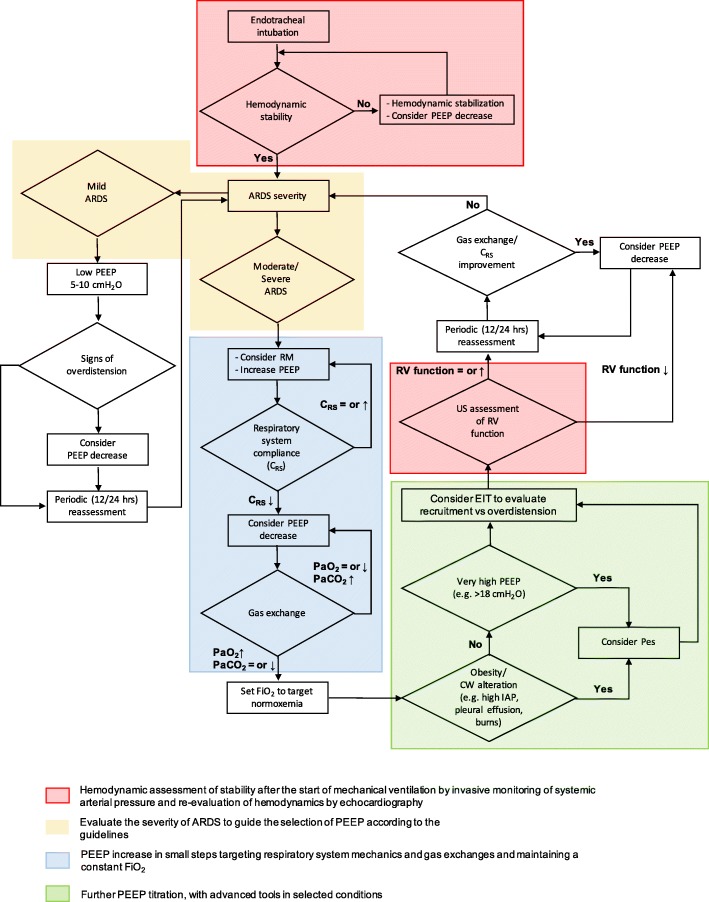
Evidence-based decision-making flow chart for patients with ARDS requiring treatment using PEEP, according to patient physiological readouts. The approach we use to set up PEEP is applied either to patients in a supine position or to those with moderate-to-severe ARDS and prone positioning. Each step lasts normally 10 to 30 min. The area in light blue indicates that FiO_2_ remains constant throughout the steps. After PEEP titration FiO_2_ can be decreased (or increased) to target normoxia. Pre-existing barotrauma and (according to some authors) elevated intracranial pressure should discourage from application of high PEEP. Abbreviations and symbols: ARDS, acute respiratory distress syndrome; C_RS_, compliance of the respiratory system; CW, chest wall; EIT, electrical impedance tomography; FiO_2_, inspiratory oxygen fraction; PEEP, positive end-expiratory pressure; Pes, esophageal pressure; RM, recruitment maneuver; RV, right ventricle; US, ultrasound; ↑, increase; ↓, decrease; =, equal

For moderate and severe ARDS, the guidelines [6] recommend higher PEEP levels without specifying absolute values or, more importantly, what methodology to apply. Therefore, for patients with moderate or severe ARDS, we typically aim to increase PEEP levels, if hemodynamic conditions allow it, through closely monitoring the individual response and focusing on two main targets: driving pressure and oxygenation (Fig. 1).

### Driving pressure

C_RS_ is proportional to the “baby lung” size [9]; therefore, as a good proxy of EELV (albeit possibly influenced by other factors, such as chest-wall compliance), C_RS_ tends to increase with recruitment but decreases again once over-inflation begins. For this reason, changes in C_RS_ are a key element for PEEP titration. At the same tidal volume (*V*_T_), any change in C_RS_ will be reflected in the driving pressure (DP) [10], or if pressure control is used, *V*_T_ increases for the same DP. We increase PEEP levels aiming to observe a decrease in DP at the same *V*_T_, likely indicating recruitment (not necessarily to a fully open lung). To facilitate this process, we could use a moderate recruitment maneuver (RM) (e.g., 40 cmH_2_O for 20 s) before increasing PEEP. An RM (rather than to correct hypoxemia) might work as a diagnostic tool (*diagnostic RM*) to explore the potential for lung recruitability, leading to an increase in PEEP levels in responders compared with non-responders. Simultaneously, if C_RS_ decreases when PEEP is increased, indicating overdistension, we reduce either PEEP or *V*_T_ (if feasible in terms of CO_2_ elimination and respiratory rate). For a safe plateau pressure (*P*_plat_), one size (i.e., 30 cmH_2_O) does not fit all, and if overdistension is an issue, our safety threshold for *P*_plat_ is decreased.

### Oxygenation

We always verify the response to gas exchange, primarily, an increase in PaO_2_ at a constant inspiratory FiO_2_, with constant or decreasing PaCO_2_. Although PaO_2_/FiO_2_ is a poor proxy for alveolar recruitment, patients who have responded to an increased PEEP with improved oxygenation have been reported to have a reduced risk of death [11]. As such, we prefer to uncouple the PEEP and FiO_2_ settings. Patients do not always show an improvement in oxygenation with higher PEEP levels. In this scenario, a strategy that mandates simultaneous increase of these parameters (e.g., PEEP/FiO_2_ tables) would recommend a further PEEP increase combined with FiO_2_. Finally, an increase in PaCO_2_ levels in relation to a PEEP increase should be an immediate alert for a risk of overdistension.

Of late, and more frequently, we are taking advantage of bedside electrical impedance tomography (EIT) to corroborate our PEEP titration procedure. We propose a 2-step strategy. First, we perform a diagnostic RM to evaluate the potential for lung recruitment. Second, we increase the PEEP level in small increments (e.g., 2 cmH_2_O) until it is sufficient to maintain EELV stability, according to the end-expiratory lung impedance signal. This approach leads to an improvement in arterial oxygenation and a reduction in the DP and provides regional information concerning the balance between alveolar overdistension and collapse [12].

We typically confine the measurement of esophageal pressure to selected clinical conditions (Fig. 1).

## Controversies concerning the use of higher PEEP levels

The described approach might appear to be contradictory to the recent literature [13] reporting that patients receiving an RM followed by a decremental PEEP trial, according to C_RS_, have increased mortality rates. However, we consider that this study does not invalidate the concept of higher PEEP levels being associated with a lower DP, as it combined other procedures that might have contributed to the higher mortality, such as an aggressive RM of up to 60 cmH_2_O (reduced to 50 cmH_2_O after 50% enrollment) and lasting several minutes overall, which required important fluid expansion, neuromuscular blocking agents, and an additional RM performed after PEEP titration. Furthermore, the decision to set PEEP at 2 cmH_2_O above the best C_RS_ likely led to regional overdistension of the non-dependent lung.

## Future perspectives and conclusion

It is known that a high PEEP level does not fit all; therefore, innovative concepts such as the different responses of hypo- and hyper-inflammatory ARDS phenotypes to PEEP [14] and the use of population enrichment for inclusion in trials [15] are encouraging.

In the meantime, we set PEEP levels for patients with moderate or severe ARDS that aim for a moderate reasonable recruitment, given the challenges of full lung recruitment, according to incremental PEEP steps (possibly interspersed with short *diagnostic RMs*) and seek improvements in functional and physiologic readouts, such as C_RS_, gas exchange, and EIT.

## Data Availability

Not applicable

## Abbreviations

ARDS: Acute respiratory distress syndrome
C_RS_: Compliance of the respiratory system
DP: Driving pressure
EELV: End-expiratory lung volume
EIT: Electrical impedance tomography
FiO_2_: Inspiratory fraction of oxygen
PaCO_2_: Arterial pressure of carbon dioxide
PaO_2_: Arterial pressure of oxygen
PEEP: Positive end-expiratory pressure
*P*_plat_: Plateau pressure
RM: Recruitment maneuver
*V*_T_: Tidal volume
